# Does dual HER-2 blockade treatment increase the risk of severe toxicities of special interests in breast cancer patients: A meta-analysis of randomized controlled trials

**DOI:** 10.18632/oncotarget.15252

**Published:** 2017-02-10

**Authors:** Shuai Hao, Wuguo Tian, Bo Gao, Yan Jiang, Xiaohua Zhang, Shu Zhang, Lingji Guo, Jianjie Zhao, Gang Zhang, Chunyan Hu, Jie Yan, Donglin Luo

**Affiliations:** ^1^ Department of Breast, Thyroid Surgery, Research Institute of Surgery, Daping Hospital, Third Military Medical University, Chongqing 400042, China

**Keywords:** dual Her-2 blockade, Her2, adverse events, breast cancer, meta-analysis

## Abstract

Although dual HER-2 blockade treatment could offer greater clinical efficacy in breast cancer, the risk of severe toxicities of special interest related to this combined regimen in breast cancer remained unknown. We systematically searched public databases (MEDLINE, EMBASE, Cochrane library) to identify relevant studies that comparing anti-HER2 monotherapy (lapatinib or trastuzumab or pertuzumab) with dual HER-2 blockade treatment (pertuzumab plus trastuzumab or trastuzumab plus lapatinib) in breast cancer. A total of 11,941 breast cancer patients from 9 trials were included for analysis. Meta-analysis showed that dual HER2 blockade treatment significantly increased the risk of severe diarrhea (OR 2.52, *p*<0.001) and treatment discontinuation (OR 1.52, *p*=0.014), but not for severe rash (OR 1.06, *p*=0.81), liver toxicities (OR 1.16, *p*=0.28), CHF (OR 1.46, *p*=0.09), LVEF decline (OR 1.09, *p*=0.40) and FAEs (OR 0.97, *p*=0.91). Similar results were observed in sub-group analysis according to anti-HER2 regimens in terms of severe diarrhea and treatment discontinuation. Additionally, trastuzumab plus lapatinib significantly increased the risk of LVEF decline in comparison with lapatinib alone (OR 1.48, *p*=0.002). Our analysis indicated that dual anti-HER2 blockade treatment significantly increased the risk of developing severe diarrhea and treatment discontinuation in comparison with anti-HER2 monotherapy. These were no evidence of an increased risk of fatal adverse events with dual-HER2 blockade treatment.

## INTRODUCTION

Approximately 15-20% of all breast cancers (HER2-positive) have human epidermal growth factor receptor 2 (HER2) protein overexpression resulting in a more aggressive phenotype and worse outcome [1–3]. The epidermal growth factor receptor (EGFR) superfamily is composed of four transmembrane tyrosine kinase receptors containing HER1, HER2, HER3, and HER4, which plays a critical role in cellular growth and proliferation [4]. HER2 is considered as an orphan receptor because it has no known ligand, and it could constitutively activate signaling pathways through ligand-independent dimerization [5]. As a result, molecular targeting of the HER2 receptor and its family members have emerged as attractive candidates for anticancer therapy [6]. Currently, two anti-HER2 agents, including humanized monoclonal antibody trastuzumab [7, 8] and the small-molecule tyrosine kinase inhibitor lapatinib [9], have been approved for use in the HER2-positive breast cancer. However, despite these treatment advances, most of HER2-positive breast cancer would eventually progress due to intrinsic and acquired HER2 agent resistance, highlighting the need to develop novel agents and combination strategies to overcome resistance.

During the past decade, a dual targeting approach by combination of two anti-HER2 agents has been investigated in several large randomized controlled trials [10]. In fact, dual anti-HER2 therapy has been proven to improve clinical outcomes of metastatic HER2 positive breast cancer though to be refractory to trastuzumab alone, which lead to the approval of pertuzumab for the treatment of HER2 positive breast cancer [11]. In addition, it has been found that dual anti-HER2 treatments almost double the rates of pathologic complete response in comparison with anti-HER2 therapy alone in the neoadjuvant setting [12, 13]. However, the safety profile of dual anti-HER2 blockade treatment remains undetermined. As a result, we perform this systematic review and meta-analysis to assess whether the dual anti-HER2 treatment would increase the risk of severe (grade 3 and 4) toxicities of special interest in breast cancer when compared to anti-HER2 monotherapy.

## RESULTS

### Search results

**Figure 1:**
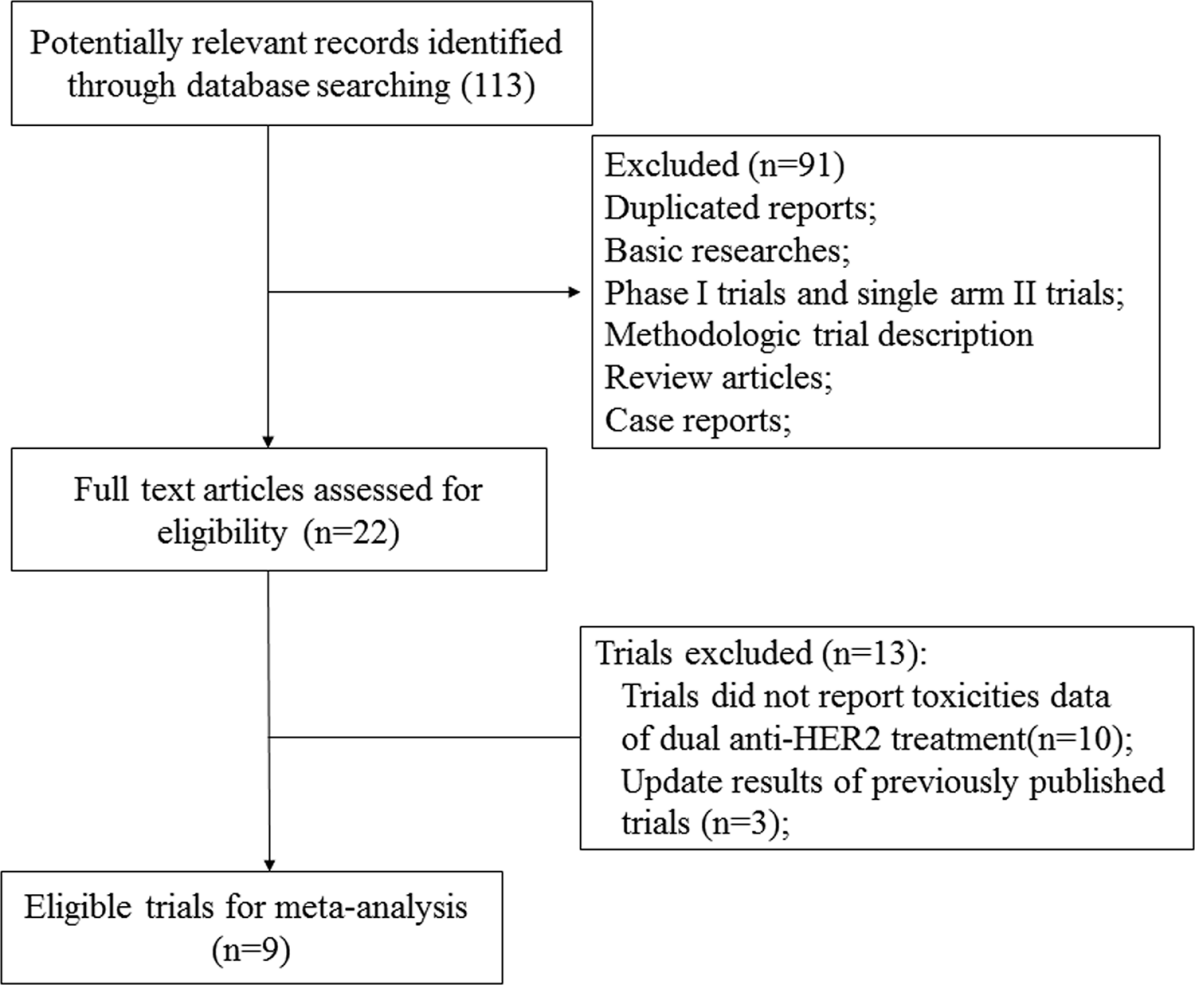
Studies eligible for inclusion in the meta-analysis

As shown in Figure 1, 22 potentially eligible trials were retrieved for full-text evaluation. The reasons for study exclusion were illustrated in Figure 1. A total of twelve trials were included for analysis [11, 12, 14–23]. Three trials were up-date results of previously published trials [16, 17, 21] and thus nine trials were finally included in this systematic review. (Figure 1) The baseline characteristics of the trials were listed in Table 1. A total of 11,701 HER2 positive breast cancer patients were included in the present study. A rough assessment of the included trials was carried out by using Jadad scale. The quality of the nine trials was high, two trials had a Jadad score of 5 [11, 15], and seven trials had a Jadad score of 3 [12, 14, 18–20, 22, 23].

**Table 1:** Baseline characteristics of nine included trials for analysis

Authors	Year	Phase	Treatment line	Total patients	Treatment regimens	Median age	Duration of anti-HER2 treatment	No. for analysis	Jadad Score
**Piccart-Gebhart M.et al**	2016	III	adjuvant	8381	L 750mg daily +T 2mg/kg weekly (loading 4mg/kg)+CT	51	52weeks	2061	3
					T 2mg/kg weekly (loading 4mg/kg)-L 750mg daily	51	12weeks-34weeks	2076	
					L 750mg daily +CT	51	52weeks	2057	
					T 2mg/kg weekly (loading 4mg/kg)+CT	51	52weeks	2076	
**Swain S.M. et al(CLEOPATRA)**	2015	III	metastatic	808	P 420mg/kg q.3.w (loading 840mg/kg)+T 6mg/kg q.3.w (loading 8mg/kg)	NR	until progression or unacceptable toxicity	369	5
					T 6mg/kg q.3.w (loading 8mg/kg)+docetaxel	NR	until progression or unacceptable toxicity	335	
**Bonnefoi H. et al**	2015	II	Neoadjuvant	128	L 1000mg daily+ CT	49.9	12weeks	23	3
					T 2mg/kg weekly (loading 4mg/kg)+CT	47	12weeks	53	
					L1000mg daily +T 2mg/kg weekly (loading 4mg/kg +CT	49.4	12weeks	52	
**Robidoux A. et al (NSABP B-41)**	2013	III	neoadjuvant	529	T 2mg/kg weekly (loading 4mg/kg)	NR	12weeks	178	3
					L 1250mg daily	NR	12weeks	173	
					T 2mg/kg weekly (loading 4mg/kg)+L 750mg daily	NR	12weeks	173	
**Guarneri V. et al (CHER-LOB)**	2012	IIb	neoadjuvant	121	T 2mg/kg weekly (loading 4mg/kg)+CT	50	26weeks	36	3
					L 1500mg daily+ CT	49	26weeks	39	
					L 100mg daily +T 2mg/kg weekly (loading 4mg/kg)+CT	49	26weeks	46	
**Gianni L. et al (NeoSphere)**	2012	II	neoadjuvant	417	T 6mg/kg q.3.w (loading 8mg/kg)+docetaxel	50	12weeks	107	3
					P 420mg/kg q.3.w (loading 840mg/kg)+T 6mg/kg q.3.w (loading 8mg/kg) +docetaxel	50	12weeks	107	
					P 420mg/kg q.3.w (loading 840mg/kg)+T 6mg/kg q.3.w (loading 8mg/kg)	49	12weeks	108	
					P 420mg/kg q.3.w (loading 840mg/kg)+docetaxel	49	12weeks	94	
**Baselga J. et al(CLEOPATRA)**	2012	III	metastatic	806	T 6mg/kg q3w (loading 8 mg/kg)+docetaxel	54	until progression or unacceptable toxicity	396	5
					P 420mg q.3.w (loading 840mg) +T 6mg/kg q3w (loading 8 mg/kg) +docetaxel	54	until progression or unacceptable toxicity	408	
**Baselga J. et al(NeoALTTO)**	2012	III	Neoadjuvant	455	L 1500mg daily+paclitaxel	50	18 weeks	154	3
					T 2mg/kg weekly (loading 4mg/kg)+paclitaxel	49	18 weeks	149	
					L1000mg daily+T 2mg/kg (loading 4mg/kg)+paclitaxel	50	18 weeks	152	
**Blackwell K.L. et al**	2010/2012	III	metastatic	296	L1,000mg daily	51	until progression or unacceptable toxicity	146	3
					L1.000mg daily+ T 2mg/kg weekly (loading 4mg/kg)	52	until progression or unacceptable toxicity	149	

Abbreviation: L, lapatinib; T, trastuzumab; P, pertuzumab; CT, chemotherapy.

### Heterogeneity

No observed heterogeneity for grade ≥3 AEs of diarrhea, liver toxicities and CHF, LVEF decline and FAEs was found (Table 2). Therefore, we pooled the risk of severe AEs related to dual anti-HER2 agents by using fixed-effect model, excepting for AEs lead to permanent treatment discontinuation and grade ≥3 rash.

**Table 2:** Peto odds ratio of adverse events related to dual anti-HER2 treatment

Adverse outcome (grade ≥3)	Trials (n)	No. of patients (n)		*I*^2^	Peto Odds Ratio (95%CI)	*p*
		Dual anti-HER2 agents, Events/total	Anti-HER2 monotherapy,Events/total			
**Diarrhea**	9	557/3649	431/5992	49%	2.52(95%CI: 2.20-2.89)	<0.001
**Rash**	9	196/3649	238/5992	61%	1.06 (95%CI:0.67-1.68)	0.81
**Liver toxicities**	6	100/2723	168/5115	43%	1.16 (95%CI:0.89-1.50)	0.28
**CHF**	9	42/3649	47/5992	46%	1.46(95%CI: 0.94-2.26)	0.09
**LVEF decline**	9	209/3649	296/5992	49%	1.09(95%CI:0.90-1.31)	0.40
**AEs lead to permanent treatment discontinuation**	9	681/3649	684/5992	74%	1.52(95%CI: 1.09-2.12)	0.014
**FAEs**	9	28/3649	39/5992	0%	0.97(95%CI: 0.59-1.59)	0.91

*I*^2^≥50% suggests high heterogeneity across studies.

Abbreviation: CHF, congestive heart failure, LVEF, left ventricular ejection fraction; FAEs, Fatal adverse events.

### AEs reported in trials and pooled effects

#### Diarrhea

**Figure 2:**
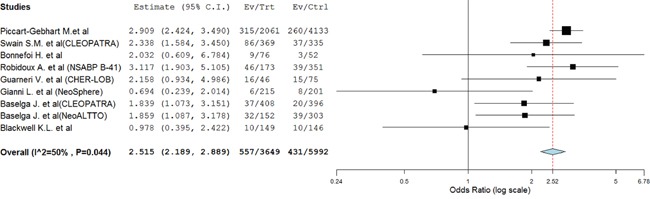
Fixed-effect Model of odds ratio (95%CI) of severe diarrhea associated with dual anti-HER2 agents versus anti-HER2 monotherapy

A total of 988 severe diarrhea were reported in the trials; 557 in dual anti-HER2 agent arms and 431 in control arms, yielding an OR of severe diarrhea of 2.52(95%CI: 2.20-2.89, *P*<0.001) (Table 2, Figure 2). Sub-group analysis also found that trastuzumab combined with lapatinib significantly increased the risk of severe diarrhea in comparison with trastuzumab (OR 6.42, 95%CI: 5.29-7.79, p<0.001) or lapatinib alone (OR 1.33, 95%CI: 1.14-1.55, p<0.001, respectively), and pertuzumab combined with trastuzumab also was associated with a significantly increased risk of severe diarrhea in comparison with trastuzumab alone (OR 2.03, 95%CI: 1.50-3.15, p<0.001, Table 3).

**Table 3:** Subgroup analyses of risk for severe adverse toxicities of special interest from dual anti-HER2 treatment

Type of anti-HER2 therapy	No. of trials	Dual anti-HER2 treatment		Monotherapy		Peto OR (95%CI)	*P* value
		Diarrhea	Total	Diarrhea	Total		
**T+L versus T**	5	418	2484	36	2492	6.42 (5.29-7.79)	<0.001
**T+L versus L**	6	428	2633	330	2592	1.33(1.14-1.55)	<0.001
**P+T versus T**	3	129	992	61	838	2.03(1.50-3.15)	<0.001
		**Rash**	**Total**	**Rash**	**Total**		
**T+L versus T**	5	117	2484	15	2492	4.88 (3.45-6.90)	<0.001
**T+L versus L**	6	150	2633	189	2592	0.76(0.61-0.95)	0.016
**P+T versus T**	3	47	992	20	838	2.09(1.27-3.44)	0.004
		**Liver toxicities**	**Total**	**Liver toxicities**	**Total**		
**T+L versus T**	5	100	2484	33	2492	2.83(2.00-4.00)	<0.001
**T+L versus L**	5	100	2484	131	2446	0.74(0.56-0.96)	0.024
**P+T versus T**	1	0	215	3	107	0.07(0.004-1.35)	-
		**CHF**	**Total**	**CHF**	**total**		
**T+L versus T**	5	24	2484	25	2492	0.97(0.55-1.70)	0.91
**T+L versus L**	6	34	2633	16	2592	1.33(0.35-5.03)	0.68
**P+T versus T**	3	7	992	7	838	0.92 (0.32-2.66)	0.88
		**LVEF decline**	**Total**	**LVEF decline**	**Total**		
**T+L versus T**	5	122	2484	124	2492	0.99(0.77-1.29)	0.96
**T+L versus L**	6	160	2633	110	2592	1.48 (1.15-1.90)	0.002
**P+T versus T**	3	49	992	63	838	0.69(0.47-1.01)	0.058
		**Treatment discontinuation**	**Total**	**Treatment discontinuation**	**Total**		
**T+L versus T**	5	565	2484	187	2492	3.31(2.83-3.87)	<0.001
**T+L versus L**	6	582	2633	384	2592	1.64(1.43-1.89)	<0.001
**P+T versus T**	3	107	992	78	838	1.01(0.47-2.18)	0.98
		**FAEs**	**Total**	**FAEs**	**Total**		
**T+L versus T**	5	9	2484	9	2492	1.01(0.40-2.54)	0.99
**T+L versus L**	6	9	2633	16	2592	0.57 (0.26-1.25)	0.16
**P+T versus T**	3	16	992	16	838	0.92(0.46-1.86)	0.83

Abbreviation: L, lapatinib; T, trastuzumab; P, pertuzumab; FAEs, fatal adverse events; CHF, congestive heart failure; LVEF, left ventricular ejection fraction.

#### Rash

Nine trials reported rash data with 196 in dual anti-HER2 arms and 238 in anti-HER2 monotherapy arms. No significantly increased risk of severe rash was detected in the anti-HER2 combination therapy with OR 1.06 (95% CI 0.67–1.68; *P*=0.81) (Table 2). Sub-group analysis showed that the combination of lapatinib with trastuzumab significantly increased the risk of severe rash in comparison with trastuzumab alone (OR 4.88, 95%CI: 3.45-6.90, *p*<0.001), while the addition of trastuzumab to lapatinib seemed to decrease the risk of developing severe rash in comparison with lapatinib alone (OR 0.76, 95%CI:0.61-0.95, *p*=0.016), which suggested that the use of lapatinib was associated with an increased risk of severe rash, and clinicians should pay attention to the risk of rash during the administration of lapatinib. Additionally, the addition of pertuzumab to trastuzumab significantly increased the risk of severe rash in comparison with trastuzumab alone (OR 2.09, 95%CI: 1.27-3.44, *p*=0.004).

#### Liver toxicities

Only six trials reported liver toxicities data with 100 patients in dual anti-HER2 agent group, and 168 in control group. We did not observe a significantly increased risk of liver toxicities with dual anti-HER2 combination regimens when compared to anti-HER2 monotherapy (OR 1.16, 95%CI: 0.89-1.50, *P*=0.28, Table 2). Subgroup analysis showed that the addition of lapatinib to trastuzumab significantly increased the risk of liver toxicities in comparison with trastuzumab alone (OR 2.83, 95%CI: 2.00-4.00, *p*<0.001). Interestingly, the addition of trastuzumab to lapatinib seemed to decrease the risk of developing severe liver toxicities when compared to lapatinib alone (OR 0.74, 95%CI: 0.56-0.96, *p*=0.024). Based on these results, clinicians should pay attention to the risk of liver toxicities during the administration of lapatinib.

### CHF and LVEF decline

**Figure 3:**
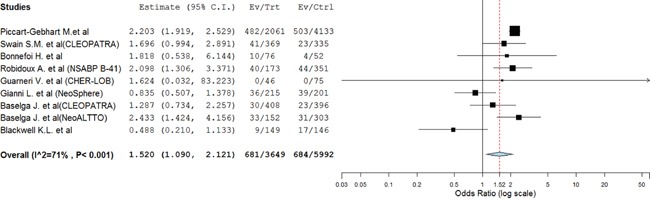
Fixed-effect Model of odds ratio (95%CI) of CHF associated with dual anti-HER2 agents versus anti-HER2 monotherapy

There were 89 CHF events was reported, with 42 in dual anti-HER2 arms and 47 in control arms. Our results found that dual anti-HER2 agents seemed to increase the risk of CHF using a fixed effect model (OR=1.46; 95% CI 0.94–2.26; *P*=0.09, Figure 3). Similar results were observed in subgroup analysis based on anti-HER2 treatment (Table 3).

A total of 9 trials reported LVEF decline data, 209 in dual anti-HER2 arms and 296 in anti-HER2 monotherapy arms. The pooled OR showed that anti-HER2 combination therapy did not increase the risk of LVEF decline (OR=1.09; 95% CI 0.90–1.31; *P*=0.40, Table 2). Subgroup analysis according to anti-HER2 regimen found that trastuzumab plus lapatinib combination therapy significantly increased the risk of LVEF decline in comparison with lapatinib alone (OR 1.48, 95%CI: 1.15-1.90; *p*=0.002, Table 3), while no significantly increased risk of LVEF decline in other dual anti-HER2 combination therapy (Table 3).

### AEs lead to permanent treatment discontinuation

**Figure 4:**
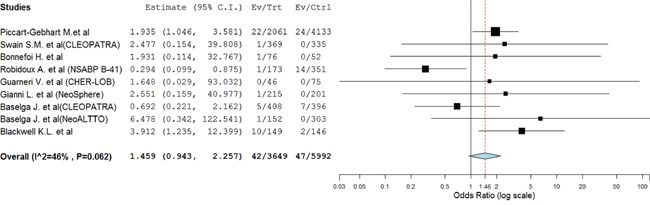
Random-effects Model of odds ratio (95%CI) of treatment discontinuation associated with dual anti-HER2 agents versus anti-HER2 monotherapy

Nine trials reported treatment discontinuation data with 681 in dual anti-HER2 arms and 684 in control arms. The pooled results showed that dual anti-HER2 combination significantly increased the risk of developing treatment discontinuation yielding OR of 1.52 (95%CI: 1.09-2.12, *p*=0.014, Figure 4). Sub-group analysis also found that trastuzumab combined with lapatinib significantly increased the risk of treatment discontinuation in comparison with trastuzumab (OR 3.31, 95%CI: 2.83-3.87, p<0.001) or lapatinib alone (OR 1.64, 95%CI: 1.43-1.89, p<0.001, Table 3).

### Fatal adverse events

A total of 289 FAEs were observed in the dual anti-HER agent group and 39 in the control group giving a pooled OR of 0.97 (95% CI 0.59–1.59; *P*=0.91, Table 2). In sub-group analysis, no increased risk of FAEs was observed in dual anti-HER2 combinations (Table 3).

### Publication bias

We did no observed publication bias for the AEs studied excepting for severe diarrhea events by Egger tests (*P*= 0.022, Table 4).

**Table 4:** publication bias Begg and Egger test (*p*-value)

	Begg	Egger
**Rash**	0.35	0.18
**Diarrhea**	0.07	0.022
**Discontinuation treatment**	0.38	0.08
**Liver toxicities**	1.0	0.64
**LVEF decline**	0.92	0.71
**CHF**	0.90	0.97
**FAEs**	0.46	0.28

## DISCUSSION

Due to the increased understanding of the molecular events involved in breast cancer development, the treatment strategy for HER2-positive breast cancer has dramatically evolved. The humanized monoclonal antibody trastuzumab has been the foundation of care for HER2 positive breast cancer in both the neoadjuvant/adjuvant and metastatic settings [24, 25]. Although the prognosis of HER2-positive breast cancer has been significantly improved by using trastuzumab, most of these patients would become resistance to trastuzumab, and new treatment strategies are clearly required. In fact, dual anti-HER2 therapy has been proven to improve clinical outcomes of early and metastatic HER2 positive breast cancer [18, 26]. However, whether dual anti-HER2 combination therapy would increase the risk of severe (grade 3 and 4) toxicities of special interest remains undetermined. In the present meta-analysis, a total of 11,941 breast cancer patients are included. Our results show that dual HER2 blockade treatment is associated with a significantly increased risk of severe diarrhea (OR 2.52, *p*<0.001) and treatment discontinuation (OR 1.52, *p*=0.014), but not for severe rash (OR 1.06, *p*=0.81), liver toxicities (OR 1.16, *p*=0.28), CHF (OR 1.46, *p*=0.09), LVEF decline (OR 1.09, *p*=0.40) and FAEs (OR 0.97, *p*=0.91).

Severe diarrhea could delay treatment or lead to permanent treatment discontinuation, which might reduce the efficacy of anti-HER2 treatment. The study of severe diarrhea events shows the highest RR with 2.52 with dual anti-HER2 treatment, is consistent with previously published meta-analyses [27]. Sub-group analysis also find that trastuzumab combined with lapatinib is associated with a significantly increased risk of severe diarrhea in comparison with trastuzumab or lapatinib alone, and pertuzumab combined with trastuzumab also significantly increases the risk of severe diarrhea in comparison with trastuzumab alone. As a result, clinicians should provide well-defined gastrointestinal monitoring during the administering of dual anti-HER2 therapy, and should provide immediate and effective management in case of severe diarrhea events.

We then assess the risk of treatment discontinuation related to dual anti-HER2 treatments. Our results show that dual anti-HER2 treatment is associated with a significantly increased risk of developing treatment discontinuation in comparison with anti-HER2 monotherapy. In sub-group analysis, we also find an increased risk of treatment discontinuation according to treatment regimens. All of the included trials except for Baselga et al’ study [11] reported the common adverse event for treatment discontinuation, which was hepatic toxicity, followed by diarrhea. Because AEs lead to permanent treatment discontinuation could reduce the efficacy of anti-HER2 treatment in breast cancer, thus clinicians should pay more attention to severe toxicities with anti-HER2 treatment.

Several previous studies have indicated a significantly increased risk of cardiac toxicities associated with anti-HER2 monotherapy trastuzumab in breast tumors [28, 29]. Wittayanukorn S et al [30] reported that trastuzumab was associated with significantly increased risk of cardiac toxicities with reporting odds ratios (ROR) as a single agent (ROR=5.74) or combination use of cyclophosphamide (ROR=16.83) or doxorubicin (ROR=17.84). Then, Valachis A et al [31] investigated the cardiac toxicities with dual HER2 blockade and did not observed an increased the risk of CHF(OR 0.58, *p*=0.17) and LVEF decline (OR 0.88, *p*=0.64) with anti-HER2 combination. In the present study, we find a tendency to increase the risk of CHF (OR=1.46; *P*=0.09) associated with dual anti-HER2 agents when compared to anti-HER2 monotherapy. Subgroup analysis shows that trastuzumab plus lapatinib combination therapy significantly increases the risk of LVEF decline when compared to lapatinib (OR 1.48, *p*=0.002), while no significantly increased risk of LVEF decline in other dual anti-HER2 combination therapy. Based on our findings, clinicians should be aware of the risk of cardiac toxicities of dual anti-HER2 treatment for the treatment of breast cancer, especially when adding trastuzumab to lapatinib.

In 2015, Abdel-Rahman O.et al performed a meta-analysis and [32] demonstrated that the use of lapatinib significantly increased the risk of skin rash in solid tumors (RR 3.04, *p*<0.001), but whether dual anti-her2 treatment would increase the risk of severe rash remains unknown. In our study, we does not observe a statistically significant increase of severe rash with dual anti-HER2 treatment in breast cancer patients, while sub-group analysis shows that lapatinib significantly increases the risk of severe rash which is consisted with previous research [36]. In addition, no increased risk of liver toxicities associated with the combination of anti-HER2 agents is detected. However, sub-group analysis shows that the addition of lapatinib to trastuzumab significantly increases the risk of liver toxicities in comparison with trastuzumab alone. Interestingly, the addition of trastuzumab to lapatinib seems to decrease the risk of developing severe liver toxicities when compared to lapatinib alone. Based on these results, clinicians should pay attention to liver toxicities during the administration of lapatinib. Additionally, we do not find a significantly increased risk of fatal adverse events associated with dual anti-HER2 treatment.

Several limitations need to be concerned in present study. First, our study is a study-level meta-analysis rather than an individual patient data, therefore a more comprehensive by confounding variables at the patient level could not be performed. Second, this study is a retrospective study, and the baseline characteristics of included study might be different, which might increase the heterogeneity between studies.

## MATERIALS AND METHODS

### Data sources

The Cochrane Central Register of Controlled Trials (CENTRAL), PubMed (up to June 2016), and Web of Science (up to June 2016) databases were searched for studies using “anti-HER2 agents”, “trastuzumab”, “pertuzumab”, “lapatinib”, “breast cancer”, “breast neoplasm”, “randomized controlled trial” and “humans”. When more than one publication was identified from the same clinical trial, we used the most recent or complete report of that trial.

To be included in the meta-analysis, a study had to satisfy the following requirements: (1) patients with pathologically confirmed breast cancer; (2) randomized controlled trials comparing anti-HER2 monotherapy (lapatinib or trastuzumab or pertuzumab) versus dual HER2 blockade treatment with or without chemotherapy regardless of treatment settings; (3) available data regarding adverse outcomes of interest (grade ≥3 AEs of diarrhea, rash, liver toxicities and congestive heart failure (CHF), left ventricular ejection fraction (LVEF) decline less than 50% or a decrease of more than 10% from baseline, AEs lead to permanent treatment discontinuation and fatal adverse events) and sample size.

### Data extraction

Two investigators independently extracted the data according to the Preferred Reporting Items for Systematic Reviews and Meta-Analyses (PRISMA) statement. A third investigator reviewed all data entries. For each study, the following information was extracted: first author's name, year of publication, trial phase, number of enrolled subjects, treatment arms, number of patients in treatment and controlled groups, median age, adverse outcomes of interest [grade ≥3 AEs of diarrhea, rash, liver toxicities and congestive heart failure (CHF), left ventricular ejection fraction (LVEF) decline, AEs lead to permanent treatment discontinuation and fatal adverse events (FAEs)], and dosage of anti-HER2 agents.

### Statistical analysis

For the calculation of odds ratio (OR), patients assigned to dual anti-HER2 treatment were compared only with those assigned to anti-HER2 monotherapy in the same trial.

We used the Peto method to calculate the pooled odds ratios (OR) with 95% confidence intervals (CIs), because this method provided the best *CI* coverage and was more powerful and relatively less biased than the fixed or random-effects analysis when dealing with low event rates. All statistical analyses were performed by using Open Meta-Analyst software version 4.16.12 (Tufts University) and Version 2 of the Comprehensive MetaAnalysis program (Biostat, Englewood, NJ).

To avoid loss of information or choices related to results from trials with multiple intervention arms (for example, three-arms trials with two anti-HER2 monotherapy arms and one combined anti-HER2 therapy arm), we merged the two relevant (anti-HER2 monotherapy) arms into one group by adding the sample sizes and numbers of people with events and we compared the merged group with the different (combined anti-HER2 therapy) arm.

Between-study heterogeneity was estimated using the χ^2^-based Q statistic [33]. Heterogeneity was considered statistically significant when *P*_heterogeneity_ < 0.05 or *I*^2^ > 50%. Potential publication biases were evaluated for severe AEs using Begg's and Egger's tests [34, 35]. A two-tailed *P* value of <0.05 without adjustment for multiplicity was considered statistically significant. The results of the meta-analysis were reported as classic forest plots. The Jadad scale was used to assess the quality of included trials based on the reporting of the studies’ methods and results [36].

## CONCLUSION

In comparison with anti-HER2 monotherapy, dual anti-HER2 blockade treatment is associated with an increased risk of developing severe diarrhea and treatment discontinuation. These are no evidence of an increased risk of fatal adverse events with dual-HER2 blockade treatment. In the appropriate clinical practice, dual HER2 blockade treatment remains justified due to its potential survival benefits.
